# Non-Hodgkin's Lymphoma Reversal with Dichloroacetate

**DOI:** 10.1155/2010/414726

**Published:** 2010-09-16

**Authors:** Dana F. Flavin

**Affiliations:** ^1^Klinik im Alpenpark, Defreggerweg 2-6, Ringsee, 83707 Tegernsee, Germany; ^2^Foundation for Collaborative Medicine and Research, 24 Midwood Drive, Greenwich, CT 06830, USA

## Abstract

In June 2007, a 48-year-old male patient, diagnosed with Stage 4 Non-Hodgkin's Follicular Lymphoma (NHL), was treated for 3 months with conventional chemotherapy resulting in a complete remission. Almost one year later tumors returned in the nasopharynx and neck lymph glands. Refusing all suggested chemotherapies, the patient began self-administering dichloroacetate (DCA) 900 mg daily with a PET scan showing complete remission four months later. Since his last PET scan, May, 2009, he remains tumor-free from continuous DCA usage.

## 1. Introduction

Non-Hodgkin's lymphoma (NHL), a cancer of the lymph system that can start anywhere in body, affects 400,000^+^ people in the United States with 66,000 new cases in 2009 [1]. NHL often presents as a low grade fever with sweating, swollen lymph nodes, general malaise, and fatigue. Although it responds well to established therapies, including chemotherapy and radiation [2], more aggressive newer treatments are being developed, including chemotherapy with whole body radiation followed by stem cell transplants [3]. While these treatments have resulted in complete remission in some patients [4], other patients, aware of the quality of life compromises sustained with aggressive therapies [3], seek alternate avenues of treatment with professionals or on their own, many of which are nonconventional or in experimental stages. One such therapy is dichloroacetate (DCA) [5].

DCA is a by-product of water chlorination [6, 7] that inhibits aerobic glycolysis. It has been used in medicine for over 30 years [8] as an investigational drug to treat severe metabolic disorders such as diabetes and hypercholesterolemia [5, 9] as well as the treatment of congenital lactic acidosis in North American children [10]. The bioavailability [11] and pharmacokinetics [12] of DCA have been well researched over several decades in adults [6], children [13, 14], and animals [15]. As a medicinal, DCA is generally well tolerated from dosages between 10 mg/Kg and 50 mg/Kg, although prolonged exposure is associated with peripheral neuropathy [16]. Its activation of the pyruvate dehydrogenase enzyme (PDH) of the mitochondria decreases glycolysis and reactivates glucose oxidation, a favorable approach to ameliorate lactic acidosis [9].

Cancer cells predominantly utilize a system of glycolysis for energy instead of the glucose oxidation used by healthy cells. Cancer appears to be a form of intracellular lactic acidosis caused by a block in the oxidation of glucose at the level of PDH (pyruvate dehydrogenase). The glycolysis metabolism of glucose increases cancer cells' lactic acid and reduces the intracellular pH [7] resulting major shifts in the intracellular biochemistry. Aerobic glycolysis, known as the “*Warburg Effect*” [17], inactivates mitochondrial respiration which allows cancer cell growth [18]. DCA reverses this glycolysis causing several major detrimental changes in the cancer tumor cells.

First and foremost DCA inhibits pyruvate dehydrogenase kinase (PDK). PDK blocks pyruvate dehydrogenase (PDH) through its phosphorylation activity. When this kinase is inhibited by DCA, the PDH is reactivated causing the mitochondria to no longer be hyperpolarized, instead the membrane and the mitochondria are depolarized, reactivating the mitochondrial K^+^ channels which then decreases cytosolic K^+^. When PDH is inhibited in cancer cells by PDK, an excess cytosolic K^+^occurs that inactivates the caspases 3 and 9, important factors in apoptosis. DCA reactivates these caspases along with an increase in H_2_O_2_ intracellularly, allowing the release of cytochrome c from the mitochondria. The release of cytochrome c is a major activating step for cell apoptosis as it triggers the caspase cascade [19]. The results of DCA on cancers are seen both in vitro and in vivo. These effects are not seen in normal cells.

Dichloroacetate's other major effect on cancer cells is the release of mitochondrial calcium (Ca^++^). The increase of Ca^++^ in cancer cells is associated with the increase and proliferation of transcription factors. Calcium also activates ornithine decarboxylase, the rate limiting enzyme in DNA synthesis [20], and the antiapoptosis factor NFAT (nuclear factor of activated T lymphocytes) [21]. When the calcium decreases with the introduction of DCA, the cell is further directed toward apoptosis and a decrease in cell replication. In addition to DCA causing a major shift in the mitochondria, cytoplasm, and cellular membrane [19], the end effect of DCA is a cell cycle arrest in the Gap 1 phase (G_1_), which also increases apoptosis [22].

## 2. Materials and Method

After being successfully treated with six treatments of Rituxan plus CHOP (cyclophosphamide, doxorubicin hydrochloride, vincristine, and prednisolone) regime over a period of three months in 2007, a positron emission tomography (PET) scan showed a complete remission of the NHL. With no further treatments by August 2008, the PET showed his tumors returned in the nasopharynx and neck lymph glands which presented with a low grade fever of 99.8, sweating and fatigue.

The Non-Hodgkin's Lymphoma patient refused conventional therapy, instead personally obtaining dichloroacetate (DCA) he began self-administering 900 mg daily at 10 mg/kg in August 2008, adding a daily 750 mg of thiamine to protect his nerves from toxicity [15, 23]. Four months later a PET scan showed complete remission (see Figure 2). He has remained tumor-free on the continued regime of DCA and thiamine since his last PET in May 2009. Monthly blood tests are showing that all of his parameters are normal.

## 3. Results

In August 2008, an NHL patient, who had been in remission for almost a year after chemotherapy, complained of soreness and tenderness in his neck area where protrusions were visible upon examination. A PET was taken to investigate the nature of the problem and the extent of lymph involvement. 

**Figure 1:**
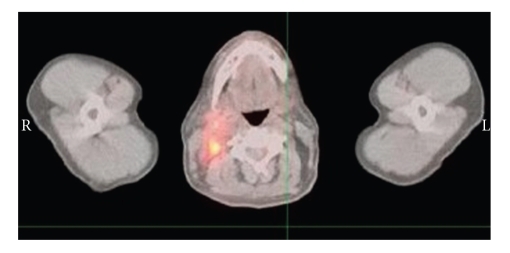
August 2008 PET scan.

Figure 1 shows that several new hypermetabolic foci within the head and neck compatible with recurrent lymphoma; new hypermetabolism in the right postlateral aspect of the nasopharynx, measuring 3.2 × 2.2 cm; new hypermetabolic adenopathy within the right neck involving the right jugulo digastric region, right jugular chain, and right posterior triangle extending to the base of the neck; the largest node measuring approximately 1.9 × 1.9 cm; several smaller hypermetabolic lymph nodes in the posterior triangle extending to the base of the neck; a single focal area of hypermetabolism within the left posterior triangle corresponding to a small lymph node which measured 1.0 × 0.5 cm.

**Figure 2:**
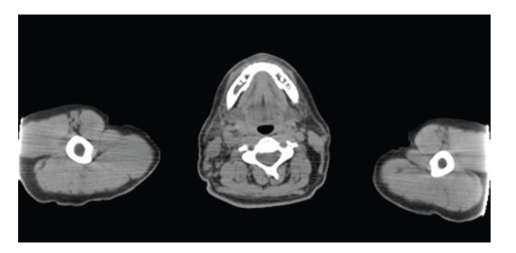
December 2008 PET scan.

Four months after the patient's daily self-medication with 750 mg of DCA, a PET scan showed no visible signs of lymphoma. Symptoms disappeared after several weeks and the results of the PET scan 4 months later in Figure 2 show that the previously seen foci of abnormal activity within the nasopharynx and neck had resolved; no abnormal foci of metabolic activity were seen; no evidence of recurrent disease.

## 4. Discussion

The medical community is seeing more and more patients who are seeking forms of therapy on their own with varying results; some are deleterious and endangering while others may prolong their lives but should still be done under medical supervision. Understandably physicians frequently cannot ethically advise or administer the use of the patents' preferences, leaving the patient to their own devices. Although this case, and others anecdotally, resulted in a successful outcome that might be explained by the existing extensive research on the pharmacology and toxicology of the dicholoroacetate treatment the patient chose, the compound's application in cancer patients is still under investigation. We are presently looking at in vitro tumor samples for testing sensitivity to DCA. We are also looking at laboratory parameters for a possible laboratory correlation in responders to specific enzyme levels as some patients' cancers respond positively or are resolved, DCA does not appear to be not tumor type specific.

Tumor cells preferentially use glycolysis to generate adenosine triphosphate (ATP) even in the presence of oxygen, a phenomenon known as aerobic glycolysis or the “*Warburg Effect*” [17]. Pyruvate dehydrogenase (PDH), a gate-keeping enzyme for the entry of pyruvate into the mitochondrial tricarboxylic acid (TCA) cycle [24], is inhibited in cancer cells by phosphorylation from the enzyme pyruvate dehydrogenase kinase (PDK) [18]. This inhibition of PDH by PDK results in a shift from glucose oxidation to glycolysis, which favors tumor growth [19]. DCA has been shown to block this phosphorylation by PDK at the mitochondrial membrane level and decrease glycolysis in favor of glucose oxidation. This return to a normal metabolism of glucose allows for major changes including a decrease in Ca^++^ intracellularly, and stabilization of the mitochondria allowing a reactivation of caspases in cancer cells leading to apoptosis [19].

The effects of DCA, caused by reactivation of mitochondrial respiration, are not without complications although it inexplicably seems to be predominantly limited to cancer cells while most normal cells remain unaffected [24]. A reversible, minimal nerve damage can be considerably reduced by a daily thiamine intake of several hundred milligrams for humans [23] and animals [15]. The thiamine amount varies from 50 mg/day to 100 mg/day depending on whether it is administered orally or injected intramuscularly [23].

Correcting mitochondrial dysfunction may be one of the major future pharmacological targets for treating many diseases, as many diseases' mitochondrial dysfunction appears to be a common pathological denominator. Lactic acidosis is also seen as a complication in malaria [25] indicating mitochondrial involvement, and more recently Chronic Fatigue Syndrome [26]. DCA has also been shown to help considerably in diabetes [27] and familial hypercholesterolemia [28].

## 5. Conclusion

A Non-Hodgkin's lymphoma patient taking 10 mg/kg [750 mg] of dichloroacetate daily of his own accord, had a complete remission of his Non-Hodgkin's lymphoma cancer after four months that has continued to date by his maintaining his DCA dosage in addition to taking 750 mg thiamine to protect against the slight tingling and numbness in the nerves of the fingers and toes, without compromising his quality of life or affecting the treatment's efficacy. Ignoring medical advice not to self-medicate he has continued his DCA/thiamine regimen, stating his concern that discontinuing DCA may allow a recurrence of the disease.

There is too little data to draw absolute conclusions on DCA's usage for cancer. Controlled research needs to be conducted for validation and confirmation of DCA's efficacy and maintenance levels in the spectrum of cancer therapies.

##  Conflict of Interests

The author reports no conflict of interests. The author alone is responsible for the content and writing of the paper.
